# N-terminal Pro-brain Natriuretic Peptide as a Prognostic Biomarker for Cardiac Surgeries: A Systematic Review

**DOI:** 10.21470/1678-9741-2024-0417

**Published:** 2025-10-31

**Authors:** Barbara Giovanna Souza Silva Queiroz, Andressa Maranhão de Arruda, Lara Maria Moura de Sá Villa-Chan, Lays Sthefany Siqueira da Costa, José Gildo de Moura Monteiro Junior, Ana Célia Oliveira dos Santos

**Affiliations:** 1 Programa de Pós-Graduação em Ciências da Saúde, Faculdade de Ciências Médicas, Universidade de Pernambuco, Recife, Pernambuco, Brasil; 2 Faculdade de Ciências Médicas, Universidade de Pernambuco, Recife, Pernambuco, Brasil

**Keywords:** Biomarkers, Cardiac Surgery, Prognosis, Systematic Review

## Abstract

**Introduction:**

N-terminal pro-brain natriuretic peptide (NT-proBNP) is a biomarker for heart stress and heart failure, with its production triggered by the stretching of cardiac fibers. This study investigates if elevated NT-proBNP levels can independently predict poor outcomes for patients undergoing heart surgery.

**Methods:**

A systematic review was performed in the PubMed®, Latin American and Caribbean Health Sciences Literature (or LILACS), Physiotherapy Evidence Database (PEDro), Web of Science, and Embase databases, with the following descriptors: "NT-proBNP" OR "NTproBNP" OR "N- terminal pro-B-type natriuretic peptide" OR "N- terminal pro brain natriuretic peptide" OR "amino terminal pro brain natriuretic peptide" AND "Cardiovascular Surgical Procedures" NOT "Pediatric" OR "children" NOT "cancer" OR "oncology" NOT "animal*". Articles that evaluated NT-proBNP and adverse outcomes in cardiac surgical patients were chosen. The levels of evidence and the strength of recommendation were assessed considering the Grading of Recommendations, Assessment, Development and Evaluation (or GRADE) system and validity by the PEDro scale. For systematic review, the Preferred Reporting Items for Systematic Reviews and Meta-Analyses (or PRISMA) criteria and the Population, Intervention, Comparison, Outcome (or PICO) strategy were followed.

**Results:**

Forty-seven articles were included, of which 17 were related to serious complications, including mortality.

**Conclusion:**

Preoperative NT-proBNP is a prognostic marker for mortality, length of stay in the postoperative intensive care unit, postoperative acute kidney injury, postoperative atrial fibrillation, postoperative low cardiac output, postoperative prolonged mechanical ventilation time, prolonged hospitalization time, unscheduled hospital readmission related to heart problems, and postoperative heart failure.

## INTRODUCTION

**Table:** 

Abbreviations, Acronyms & Symbols				
AF	= Atrial fibrillation		LCO	= Low cardiac output
AKI	= Acute kidney injury		LILACS	= Latin American and Caribbean Health Sciences Literature
AMI	= Acute myocardial infarction		LVEF	= Left ventricular ejection fraction
AS	= Aortic stenosis		MV	= Mechanical ventilation
AVR	= Aortic valve replacement		MVS	= Mitral valve surgery
BNP	= Brain natriuretic peptide		NA	= Not available
CABG	= Coronary artery bypass grafting		NNE	= Northern New England
CAD	= Coronary artery disease		NT-proBNP	= N-terminal pro-brain natriuretic peptide
CCE	= Cardiac cycle efficiency		NYHA	= New York Heart Association
CI	= Confidence interval		OR	= Odds ratio
CRP	= C-reactive protein		PEDro	= Physiotherapy Evidence Database
ECC	= Extracorporeal circulation		PMVR	= Percutaneous mitral valve repair
EuroSCORE	= European System for Cardiac Operative Risk Evaluation		PSHF	= Postoperative severe heart failure
GRADE	= Grading of Recommendations, Assessment, Development and Evaluation		RSV SAVR	= Rupture of the ventricular septum = Surgical replacement of the aortic valve
HF	= Heart failure		SR	= Sinus rhythm
HTx	= Heart transplantation		SVR	= Surgical ventricular remodeling
IABP	= Intra-aortic balloon pump		TAVR	= Transcatheter aortic valve replacement
ICU	= Intensive care unit		TMVR	= Transcatheter mitral valve repair

The cerebral N-terminal pro-brain natriuretic peptide (NT-proBNP) is a precursor of brain natriuretic peptide (BNP) hormone, which is produced and released by ventricular cardiomyocytes in response to myocardial wall stress and ischemia^[1-4]^. Elevations in NT-proBNP levels have been associated with poor outcomes in a variety of settings, including acute coronary syndrome, congestive heart failure (HF), and major noncardiac surgery^[5-7]^. In patients with asymptomatic and symptomatic aortic stenosis (AS), NT-proBNP is independently associated with outcomes^[8]^. NT-proBNP is also a predictor of outcomes after valve replacement surgery in AS^[8,9]^. Several studies have recently investigated associations of NT-proBNP with outcomes after transcatheter aortic valve replacement^[10]^.

Preoperative assessment systems in cardiac surgery patients like the European System for Cardiac Operative Risk Evaluation (or EuroSCORE) have been widely used to predict the risk of postoperative mortality. However, these systems are limited by their complexity, subjectivity in calculation, and suboptimal performance in predicting worse postoperative morbidity. In addition, they may not apply to all patient cohorts^[11,12]^.

With the increasing number of patients undergoing high-risk cardiac surgeries, accurate risk assessment becomes crucial for clinical management and the implementation of preventive measures^[13,14]^. We assume that NT pro-BNP is an independent predictor of adverse outcomes. To investigate this hypothesis, we conducted a systematic review.

## METHODS

A systematic literature review relating to NT-proBNP and cardiac surgical patients was conducted. The PubMed®, Latin American and Caribbean Health Sciences Literature (or LILACS), Physiotherapy Evidence Database (PEDro), Web of Science, and Embase databases were used. The descriptors used to search all databases were: "NT-proBNP" OR "NTproBNP" OR "N- terminal pro-B-type natriuretic peptide" OR "N- terminal pro brain natriuretic peptide" OR "amino terminal pro brain natriuretic peptide" AND "Cardiovascular Surgical Procedures" NOT "Pediatric" OR "children" NOT "cancer" OR "oncology" NOT "animal*".

The final PubMed® search strategy used as a basis for the other databases was: ((((((((((NT-proBNP) OR (NTproBNP)) OR (N- terminal pro-B-type natriuretic peptide)) OR (N- terminal pro brain natriuretic peptide)) OR (amino terminal pro brain natriuretic peptide))) AND (Cardiovascular Surgical Procedures)) NOT ((Pediatric) OR (children))) NOT ((cancer) OR (oncology))) NOT (animal*)).

The parameters adopted for inclusion and exclusion in this research were addressing a theme appropriate to the one presented here, reporting a study on human beings, containing clear, objective principles consistent with the title of the research, observational studies, and retrospective and prospective cohorts that investigated the association between preoperative NT-proBNP and postoperative complications, including death, in adults and elderly patients who underwent cardiac surgeries such as myocardial coronary artery bypass grafting (CABG), valve replacements or repairs, as well as tumor resections and heart transplantation. Articles that did not meet these criteria were not selected, such as studies with percutaneous coronary intervention or angioplasty, large-vessel surgeries, noncardiac surgeries, randomized studies, and reviews. Initially, the studies were selected by title and abstract; only when there was not enough information in the title and abstract to allow a clear decision, the studies were obtained in full.

All selected studies were evaluated in full to obtain essential information. Levels of evidence and strength of the recommendation were assessed considering the Grading of Recommendations, Assessment, Development and Evaluation (GRADE) system and validity by the PEDro scale. A systematic review was conducted following Preferred Reporting Items for Systematic Reviews and Meta-Analyses (or PRISMA) guidelines and a Population, Intervention, Comparison, Outcome (or PICO) strategy. No time filter was applied; however, data extraction took place up to January 2023. This review was registered on the PROSPERO platform under registration number CRD42023435271.

Data were extracted from the selected articles, including the first author's last name, publication year, study period, number of cases and participants, primary and secondary outcomes, objectives, and key results. A comparison across databases was conducted to identify and eliminate any duplicate studies.

## RESULTS

**Fig. 1:**
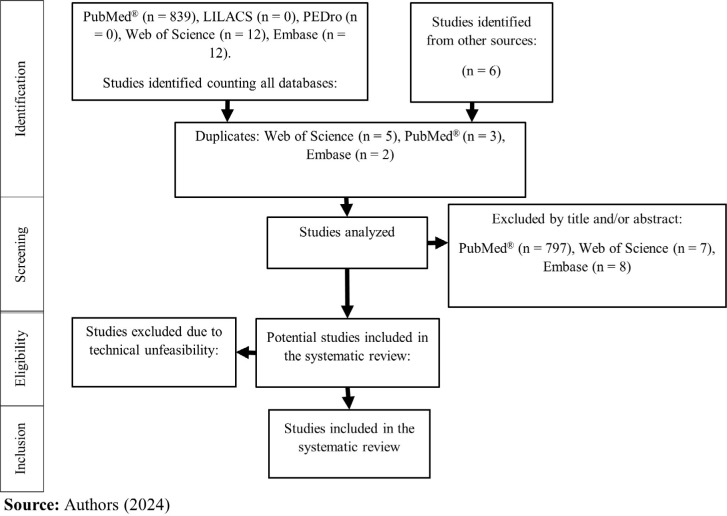
Study selection flowchart. LILACS=Latin American and Caribbean Health Sciences Literature; PEDro=Physiotherapy Evidence Database.

An initial literature search identified 869 records. After screening and abstract review, 52 studies were selected for full-text evaluation. Following a thorough assessment, three studies were excluded due to technical infeasibility, and six were excluded as they originated from sources other than peer-reviewed journals (Figure 1). Ultimately, 47 studies met the predefined inclusion criteria and were included in this systematic review.

In total, 58,743 patients underwent heart surgeries in our survey, of which 29.8% had only CABG^[15-28]^, 21.3% had only had valve surgeries^[29-38]^, 4.3% only had ventricular remodeling^[39,40]^, 2.1% had a heart transplant^[41]^, 2.1% had septal myectomy^[42]^, and 40.4% had more than one modality of cardiac surgery^[3,43–60]^.

Mortality was the most frequent outcome, reported in 33 studies^[3,15,17,19,22-24,28-34,36-42,44,45,48-54,56,58,60]^; other outcomes were prolonged postoperative intensive care unit (ICU) stay (16 studies^[15,19,22,23,28,31,34,43,44,48,50–52,54,58,60]^), postoperative acute kidney injury (AKI) (14 studies^[3,22,23,28,34,43–45,50,54,57–60]^), postoperative low cardiac debit (14 studies^[3,15,18,22,23,43,44,48,50,52,54,55,58,60]^), postoperative new atrial fibrillation (AF) (14 studies^[3,15,16,19–21,23,25,27,28,34,36,48,52]^), prolonged postoperative duration of mechanical ventilation (MV) (nine studies^[15,22,28,43,44,48,51,52,54]^), postoperative cerebrovascular events (nine studies^[3,22,23,28,36,40,44,51,58]^), prolonged length of hospital stay (eight studies^[15,19,21,31,42,48,54,60]^), unscheduled hospital readmission related to heart problems (four studies^[19,37,38,58]^), emergency reoperation for bleeding (four studies^[3,28,44,58]^), postoperative acute myocardial infarction (AMI) (three studies^[15,22,28]^), postoperative HF (three studies^[37,39,51]^), postoperative infection (three studies^[3,28,58]^), and presence of postoperative delirium (two studies^[22,46]^) (Tables 1 and 2).

**Table 1:** Characteristics of the included studies.

Author	Sample	Age (years)	Sample characteristic	Objective
ABDEL-ALEEM et al.^[15]^, 2021	65	57.62, ± 7.21	CABG	To study the role of preoperative NT-proBNP level as a predictor of adverse postoperative outcomes and in-hospital mortality.
AKHMEDOVA et al.^[43]^, 2020	28 (adults)	Group 1: 58.00, ± 12.17 Group 2: 61.46, ± 6.32	Cardiac surgery	To define the relationship of preoperative NT-proBNP testing in routine cardiac surgery practice with clinical and perioperative variables, surgical outcomes, and complications in pediatric and adult cardiac surgery. Furthermore, to evaluate the relationship between NT-proBNP and EuroSCORE II in adult patients undergoing cardiac surgery.
ANANTHA-NARAYANAN et al.^[29]^, 2019	222	78.00, ± 8.00	TAVR	To analyze the impact of baseline NT-Pro BNP and pre-TAVR diastolic parameters on long-term survival and quality of life measures in patients undergoing TAVR.
ARRIBAS-LEAL et al.^[16]^, 2007	102	AF: 67.70, ± 8.80 No AF: 63.90, ± 9.40	CABG	To determine whether the onset of postoperative AF in patients undergoing CABG was associated with preoperative plasma concentrations of CRP and NT-proBNP, preoperative statin treatment, and the use of ECC.
BALLOTTA et al.^[44]^, 2010	31	62.00, 56.00 – 71.00	Cardiac surgery	To assess whether SVR results in an immediate and significant decrease in postoperative NT-proBNP over time and whether preoperative NT-proBNP and changes in its postoperative value are associated with morbidity and mortality in patients undergoing SVR.
BARBIERI et al.^[30]^, 2019	3595	77.00, 70.00 – 82.00	TAVR e SAVR	To assess the role of plasma troponin levels in patients with severe aortic stenosis.
BELLEY-CÔTÉ et al.^[45]^, 2016	960	70.70, ± 10.40	Cardiac surgery	To assess whether cardiac biomarkers were associated with severe AKI, defined as doubling of serum creatinine or need for renal replacement therapy during hospital stay after surgery, and mortality.
BROWN et al.^[17]^, 2010	1731	NA	CABG	Evaluating whether preoperative biomarkers reflecting myocardial damage, inflammation, and metabolic dysfunction are associated with an increased risk of mortality after CABG and the use of biomarkers associated with these lesions will improve the NNE coronary artery bypass graft mortality risk prediction model.
BURKE et al.^[31]^, 2018	142	79.00, 74.00 – 86.00	TAVR	To assess whether acute HF at the time of TAVR increases mortality.
CAI et al.^[46]^, 2020	635	57.42, ± 12.69	Cardiac surgery	To assess the relationship between delirium and cardiac function.
CASTELVECCHIO et al.^[39]^, 2018	143	65.00, 58.00 – 71.00	SVR	Prospectively investigating if the longitudinal profile of serial assessments of NT-proBNP levels in patients with ischemic HF undergoing SVR and with NT-proBNP levels at different time points are associated with the outcome.
CHEN et al.^[18]^, 2007	52	Group A: 59.50 ± 10.50 Group B: 60.61 ± 10.39	CABG	This study aimed to evaluate the relationship between the preoperative level of NT-proBNP and the need for inotropic support in the immediate postoperative period of patients undergoing CABG.
CESARI et al.^[47]^, 2008	92	72.50, 47.00 – 88.00	Cardiac surgery	To evaluate the role of inflammatory mediators in influencing the number of circulating endothelial progenitor cells in patients undergoing cardiac surgery.
CHEN et al.^[19]^, 2013	76	64.00, ± 10.20	CABG	To clarify the relationship between serum B-type natriuretic peptide and NT-proBNP with the clinical course of the patient.
CUTHBERTSON et al.^[49]^, 2013	1.010	66.00, 22.00 – 89.00	Cardiac surgery	To assess the ability of NT-proBNP to predict 3-year mortality compared to validated clinical risk prediction tools.
CUTHBERTSON et al.^[48]^, 2009	1.010	66.00, 22.00 – 89.00	Cardiac surgery	To evaluate the ability of NT-proBNP to predict early postoperative outcomes of patients undergoing cardiac surgery.
ELÍASDÓTTIR et al.^[50]^, 2008	135	67.00, 56.00 - 88.00	Cardiac surgery	To determine whether postoperative complications after cardiac surgery were correlated with elevated preoperative serum NT-proBNP levels and to compare the utility of serum NT-proBNP, ejection fraction assessed by transesophageal echocardiography, and EuroSCORE as predictors of complications after cardiac surgery.
GASPAROVIC et al.^[20]^, 2010	215	SR: 60.00, ± 9.00 AF:66.00, ± 7.00	CABG	To evaluate the clinical utility of NT-proBNP fragment, troponin T, transcoronary lactate gradient, and CRP as predictors of AF in patients undergoing CABG treatment alone.
GIBSON et al.^[21]^, 2009	275	65.00, 58.00 - 70.00	CABG	To prospectively compare the ability of echocardiographic parameters and cardiac neurohormones, BNP, and NT-proBNP to predict AF in this setting.
HOLM et al.^[22]^, 2013	383	68.00, ± 9.00	CABG	To evaluate the predictive value of NT-proBNP in patients with acute coronary syndrome undergoing CABG.
HOLM et al.^[23]^, 2014	365	68.00, ± 9.00	CABG	To assess whether preoperative NT-proBNP could provide additional prognostic information to EuroSCORE II.
HUNG et al.^[24]^, 2021	71	68.68, ± 9.28	CABG	To compare a wide range of preoperative, intraoperative, and postoperative parameters between patients with in-hospital mortality and patients with in-hospital survival and to investigate risk factors for in-hospital mortality in patients undergoing emergency CABG.
ISKESEN et al.^[25]^, 2011	117	Yes: 69.00, ± 7.02 No: 59.60, ± 10.20	CABG	To assess whether preoperative and postoperative NT-proBNP levels are predictors of postoperative paroxysmal AF in patients undergoing CABG.
ISLAMOGLU et al.^[26]^, 2008	30	60.12, ± 8.77	CABG	To evaluate the diagnostic performance and prognostic significance of the NT-proBNP test in the evaluation of postoperative left ventricular diastolic dysfunction in patients undergoing CABG, comparing the NT-proBNP with the gold-standard echocardiographic results of the same patients.
JIANG et al.^[51]^, 2018	2978	70.00, 63.00 - 76.00	Cardiac surgery	To investigate the role of underlying heart disease on preoperative NT-proBNP levels in patients admitted for adult cardiac surgery, after adjusting for known confounders: age, sex, obesity, and renal function. The second objective was to investigate the predictive value of preoperative NT-proBNP about severe postoperative HF and postoperative mortality.
JOGIA et al.^[52]^, 2007	118	64.00, ± 9.00	Cardiac surgery	To determine the pattern of NT-proBNP secretion pre- and post-cardiac surgery, and then to investigate the correlation between serum NT-proBNP levels and postoperative clinical and biochemical outcomes.
KOMODA et al.^[41]^, 2009	72	INC*: 57.30, 44.10 - 62.40 NON**: 54.70, 42.90 - 59.50	HTx	To assess whether the pre-HTx value of NT-proBNP can be used as a prognostic marker to estimate survival after urgent HTx in critically ill patients.
KREUSSER et al.^[32]^, 2019	174	75.20, 64.90 - 81.00	PMVR	To assess whether invasive hemodynamics, echocardiographic parameters, and biomarkers predict outcomes after PMVR in patients with severe HF.
LINDMAN et al. ^[33]^, 2015	345	78.00, ± 11.00	SAVR e TAVR	To determine whether multiple biomarkers of cardiovascular stress are associated with mortality in patients with AS undergoing AVR regardless of clinical factors.
LINDMAN et al.^[53]^, 2018	665	71.00, 63.00 - 77.00	Cardiac surgery	To assess whether a multi-marker approach can identify patients with higher mortality and hospitalization rates after aortic valve replacement for AS.
LIU et al.^[54]^, 2013	225	61.25, ± 12.54	Cardiac surgery	To find out the factors that influence plasma levels of NT-proBNP, and then to assess whether preoperative plasma levels of NT-proBNP could predict postoperative outcomes of cardiac surgery.
MATSUURA et al.^[27]^, 2013	100	SR: 66.70, ± 8.50 AF: 70.80, ± 8.70	CABG	To assess whether NT-proBNP can predict the incidence of AF after off-pump CABG.
PASERO et al. ^[55]^, 2021	55	72.00, 60.00 – 78.00	Cardiac surgery	To estimate the incidence of vasoplegia in a homogeneous cohort of non-severe heart disease patients, to define the role of preoperative adrenal insufficiency, and to evaluate the trends of copeptin and NT-proBNP in the perioperative period.
PERREAS et al.^[34]^, 2014	75	64.80 ± 10.38	MVS	To investigate whether immediate pre and postoperative serial measurements of NT-proBNP can serve as surrogate markers of the severity status of these surgical patients and predictors of their immediate postoperative progress.
POLINENI et al.^[3]^, 2018	1.554	Live: 65.20, ± 10.10 Deceased: 70.20, ± 10.70	Cardiac surgery	To assess whether galectin-3, NT-Pro BNP, and ST2 soluble can improve the predictive ability of an existing prediction model of mortality.
RAMKUMAR et al.^[56]^, 2019	1648	65.00 ± 10.10	Cardiac surgery	To explore the relationship between long-term survival after cardiac surgery and serum levels of soluble ST2 and NT-proBNP.
SCHACHNER et al.^[28]^, 2010	819	Discharge alive: 67.00, 27.00 – 89.00 Discharge dead: 76.00, 55.00 – 80.00	CABG	To determine the influence of preoperative serum NT-proBNP on postoperative outcome and medium-term survival in patients undergoing CABG.
SCOLLETTA et al.^[35]^, 2010	25	71.50, ± 6.20	SAVR	To investigate the relationship between NT-proBNP and CCE values in patients with AS undergoing cardiac surgery for AVR.
SONG et al.^[42]^, 2019	758	46.10, ± 13.80	Septal myectomy	To determine the prognostic value of NT-proBNP in these patients.
SPAMPINATO et al.^[36]^, 2020	499	68.00, ± 9.00	SAVR	To investigate whether a combination of biomarkers related to cardiovascular stress, inflammation, and damage is associated with mortality in patients with severe AS undergoing AVR.
TANAKA et al.^[37]^, 2021	485	76.80, ± 9.20	TMVR	To investigate the association of periprocedural changes in NT-proBNP levels with clinical outcomes after edge-to-edge TMVR.
VERWIJMEREN et al.^[57]^, 2021	539	75.00, 72.00 – 77.00	Cardiac surgery	To evaluate the association between preoperative biomarkers reflecting cardiac, inflammatory, renal, and metabolic disorders and AKI associated with cardiac surgery in elderly patients.
VIKHOLM et al.^[58]^, 2014	390	1^st^ quartile: 63.00, ± 9.00, 2^nd^ quartile: 68.00, ± 9.00, 3^rd^ quartile: 71.00 ± 9.00, 4^th^ quartile: 73.00, ± 9.00	Cardiac surgery	To investigate whether preoperative NT-proBNP can predict postoperative NYHA functional class and hospital readmission, as well as morbidity and mortality.
WANG et al.^[59]^, 2021	35337	58.00, ± 11.00	Cardiac surgery	To study whether preoperative NT-proBNP concentration is associated with kidney injury after major cardiac surgery.
WEBER et al.^[38]^, 2006	102	69.00, ± 10.00	SAVR	To evaluate the prognostic value of NT-proBNP in patients with AS undergoing conservative treatment or AVR.
WOZOLEK et al.^[60]^, 2022	250	70.00, 64.00 - 78.00	Cardiac surgery	To assess whether cardiac biomarkers also help to better predict morbidity in the short term.
ZHAO et al.^[40]^, 2022	45	63.58, ± 8.21	Surgical repair of RSV	To analyze survival and risk factors associated with surgical treatment of RSV after AMI.

* Increase of 20% or more in maximal NT-proBNP value after urgency listing^[41]^

** No increase of 20% or more in maximal NT-proBNP value after urgency listing^[41]^

AF=atrial fibrillation; AKI=acute kidney injury; AMI=acute myocardial infarction; AS=aortic stenosis; AVR=aortic valve replacement; BNP=brain natriuretic peptide; CABG=coronary artery bypass grafting; CCE=cardiac cycle efficiency; CRP=C-reactive protein; ECC=extracorporeal circulation; EuroSCORE=European System for Cardiac Operative Risk Evaluation; HF=heart failure; HTx=heart transplantation; MVS=mitral valve surgery; NA=not available; NNE=Northern New England; NT-proBNP=N-terminal pro-brain natriuretic peptide; NYHA=New York Heart Association; PMVR=percutaneous mitral valve repair; RSV=rupture of the ventricular septum; SAVR=surgical replacement of the aortic valve; SR=sinus rhythm; SVR=surgical ventricular remodeling; TAVR=transcatheter aortic valve replacement; TMVR=transcatheter mitral valve repair

**Table 2:** Main outcomes of the included studies.

Author	Main outcomes
ABDEL-ALEEM et al.^[15]^, 2021	NT-proBNP had no significant correlation with low postoperative cardiac output (*P* = 0.168), atrial fibrillation (*P* = 0.462), postoperative myocardial infarction (*P* = 0.397), ICU length of stay (*P* ≥ 0.050), prolonged mechanical ventilation (*P* = 0.121), length of hospital stay (*P* ≥ 0.050), as well as in-hospital mortality after surgery (*P* = 0.306).
AKHMEDOVA et al.^[43]^, 2020	NT-proBNP was associated with higher surgical risk (*P* = 0.008), estimated glomerular filtration rate (*P* = 0.036), worsening of renal function (*P* = 0.049), and need for inotropic support after surgery (*P* = 0.006).
	There was no significant association with length of ICU stay (*P* = 0.817) or duration of mechanical ventilation (*P* = 0.840).
ANANTHA-NARAYANAN et al.^[29]^, 2019	NT-proBNP was associated with long-term mortality (*P* = 0.050).
ARRIBAS-LEAL et al.^[16]^, 2007	NT-proBNP was not associated with postoperative paroxysmal atrial fibrillation (*P* = 0.576).
BALLOTTA et al.^[44]^, 2010	NT-proBNP was associated with longer duration of mechanical ventilation (*P* = 0.013), ICU stay (*P* = 0.003), low cardiac output (*P* = 0.027), acute renal failure (*P* = 0.072), need for intra-aortic balloon pump (*P* = 0.072), and higher morbidity in the postoperative period (0.001).
	NT-proBNP was not associated with sepsis (*P* = 0.232), cerebrovascular events (*P* = 0.388), reoperation (*P* = 0.232), and mortality (*P* = 0.232).
BARBIERI et al.^[30]^, 2019	NT-proBNP was independently associated with mortality (*P* = 0.012).
BELLEY-CÔTÉ et al.^[45]^, 2016	NT-proBNP was independently associated with severe acute kidney injury (*P* = 0.030) and mortality (*P* < 0.001).
BROWN et al.^[17]^, 2010	NT-proBNP was independently associated with postoperative mortality (*P* = 0.006).
BURKE et al.^[31]^, 2018	NT-proBNP was associated with increased ICU stay > 24 hours (*P* < 0.001).
	NT-proBNP was not associated with total length of hospital stay > 3 days (*P* = 0.200), severe complication, or 30-day mortality (*P* = 0.595).
CAI et al.^[46]^, 2020	NT-proBNP was associated with postoperative delirium (*P* = 0.033).
CASTELVECCHIO et al.^[39]^, 2018	NT-proBNP was associated with a 1.5% increase in the risk of readmission for HF and a 4.2% increase in the risk of death.
	It had an independent association with mortality (*P* ≤ 0.001) and postoperative HF (*P* = 0.003).
CHEN et al.^[18]^, 2007	NT-proBNP was associated with the use of inotropic drugs (*P* < 0.001).
CESARI et al.^[47]^, 2008	NT-proBNP was negatively associated with preoperative and postoperative LVEF (*P* = 0.030).
CHEN et al.^[19]^, 2013	Preoperative NT-proBNP was not significantly associated with prolonged ICU stay and hospitalization (*P* = 0.230), nor with new-onset atrial fibrillation, ventricular tachycardia, ventricular fibrillation, need for intra-aortic balloon pump support, unscheduled cardiac readmission, and late cardiac mortality at 1 year (*P* = 0.140).
CUTHBERTSON et al.^[49]^, 2013	NT-proBNP was associated with 3-year mortality (*P* < 0.001) but lost effect in the multivariate analysis (*P* = 0.800).
CUTHBERTSON et al.^[48]^, 2009	NT-proBNP was associated with the need for postoperative inotropes > 24 hours (*P* = 0.001), the need for ventilation > 24 hours after surgery (*P* = 0.001), and postoperative atrial fibrillation (*P* = 0.020).
	Postoperative ICU stay > 1 day (*P* = 0.003), hospital stay > 1 week (*P* = 0.005), and 30-day mortality (*P* = 0.004) were independently associated.
ELÍASDÓTTIR et al.^[50]^, 2008	Preoperative NT-proBNP was significantly associated with ICU length of stay of > 2 days or death before the 28^th^ postoperative day (*P* < 0.001), need for inotropic agents (*P* < 0.001), or insertion of IABP (*P* = 0.001), or developed renal failure (*P* < 0.001) postoperatively. In addition, the biomarker was negatively associated with ejection fraction (*P* = 0.001).
GASPAROVIC et al.^[20]^, 2010	Preoperative NT-proBNP was associated with atrial fibrillation (*P* < 0.001).
GIBSON et al.^[21]^, 2009	NT-proBNP was associated with length of hospital stay (quartile 4, *P* = 0.010 and quartile 1, *P* = 0.070) and was independently associated with atrial fibrillation (*P* = 0.003).
HOLM et al.^[22]^, 2013	NT-proBNP was associated with ICU stay > 48 hours (*P* < 0.001), renal dysfunction (*P* < 0.001), ventilatory treatment (*P* = 0.009), and cerebrovascular events (*P* = 0.010). It had an independent association with mortality (*P* = 0.004), and low cardiac output (*P* = 0.004).
	NT-proBNP was not associated with perioperative myocardial infarction (*P* = 0.130), 30-day mortality (*P* = 0.220), and postoperative delirium (*P* = 0.160).
HOLM et al.^[23]^, 2014	EuroSCORE < 2: NT-proBNP was not associated with mortality (*P* = 1,000), nor with new atrial fibrillation (*P* = 0.710), renal failure (*P* = 0.270), or longer ICU stay (*P* = 1,000) in this group.
	EuroSCORE 2-10: NT-proBNP was associated with renal failure (*P* = 0.026), longer ICU stay (*P* = 0.002), and cerebrovascular events (*P* = 0.027); NT-proBNP was not associated with mortality (*P* = 0.080), nor with new atrial fibrillation (*P* = 0.770).
	NT-proBNP also had an independent association with low cardiac output (*P* = 0.049) and 1-year mortality (*P* = 0.014).
HUNG et al.^[24]^, 2021	NT-proBNP was associated with in-hospital mortality (OR: 1.0004, 95% CI: 1.00002 – 1.0008).
ISKESEN et al.^[25]^, 2011	NT-proBNP was associated with atrial fibrillation (*P* < 0.050).
ISLAMOGLU et al.^[26]^, 2008	Preoperative NT-proBNP was significantly related to preoperative mitral early transmitral-to-early diastolic annular velocity ratio (E/Ea) (*P* < 0.001).
JIANG et al.^[51]^, 2018	Elevated NT-proBNP was associated with mechanical ventilation time (*P* < 0.001) and independently associated with postoperative mortality (*P* = 0.014), length of ICU stay (*P* = 0.001), and postoperative heart failure (*P* = 0.001).
	There was no association with postoperative cerebrovascular events (*P* = 1,000).
JOGIA et al.^[52]^, 2007	Preoperative NT-proBNP was significantly related to ICU length of stay (*P* = 0.001), new atrial fibrillation (*P* = 0.010), mechanical ventilation time (*P* = 0.015), and use of inotropes (*P* = 0.003).
	It was not associated with mortality (*P* > 0.050).
KOMODA et al.^[41]^, 2009	Higher NT-proBNP was associated with a 30-day mortality rate after heart transplantation (*P* = 0.013).
KREUSSER et al.^[32]^, 2019	NT-proBNP was independently associated with mortality (*P* = 0.002).
LINDMAN et al.^[33]^, 2015	NT-proBNP was independently associated with a higher risk of mortality after valve replacement (*P* = 0.017).
LINDMAN et al.^[53]^, 2018	NT-proBNP was not associated with all-cause mortality (*P* = 0.560).
LIU et al.^[54]^, 2013	NT-proBNP was associated with composite clinical outcomes (use of high doses of inotropic agents or intra-aortic balloon ≥ 24 hours; elevated creatinine level for hemodialysis; cardiac events; ICU stay ≥ 5 days; dependence on ventilation ≥ 72 hours; deaths within 30 days of surgery) (*P* = 0.016).
	It showed an independent association with prolonged ventilation time (*P* = 0.009), length of ICU stay (*P* = 0.004), length of hospital stay (*P* = 0.019), and mortality (*P* = 0.008).
MATSUURA et al.^[27]^, 2013	NT-proBNP was associated with atrial fibrillation (*P* = 0.006).
PASERO et al.^[55]^, 2021	NT-proBNP was associated with post-cardiotomy vasoplegic syndrome (*P* = 0.003).
PERREAS et al.^[34]^, 2014	NT-proBNP was associated with an ideal postoperative clinical outcome (*P* < 0.001). The composite outcome was associated with mortality, prolonged ICU stay, acute kidney injury, and new atrial fibrillation (*P* = 0.030).
POLINENI et al.^[53]^, 2018	There was a significant independent association between in-hospital mortality and NT-proBNP (*P* = 0.027).
	NT-proBNP was associated with a new development of atrial fibrillation (95% CI: 1,020 – 1,130), new dialysis requirement (95% CI: 1,240 – 1,630), postoperative cerebrovascular event (95% CI: 1,110 – 1,300), low cardiac output (95% CI: 1,140 – 1,310), pneumonia (95% CI: 1,070 – 1,250), and mediastinitis (95% CI: 1,050 – 1,440).
	It was not associated with bleeding (95% CI: 0.740 – 1.100).
RAMKUMAR et al.^[56]^, 2019	Elevated NT-proBNP levels were independently associated with poorer survival (*P* = 0.001).
SCHACHNER et al.^[28]^, 2010	NT-proBNP was associated with prolonged ICU time (*P* = 0.001), acute kidney injury (*P* = 0.001), new atrial fibrillation (*P* = 0.031), duration of mechanical ventilation (*P* = 0.005), and independently associated with in-hospital mortality (*P* = 0.025).
	There was no association with cerebrovascular events (*P* = 0.119), reoperation due to bleeding (*P* = 0.761), acute myocardial infarction (*P* = 0.458), or infection (*P* = 0.745).
SCOLLETTA et al.^[35]^, 2010	NT-proBNP was associated with the severity of left ventricular dysfunction (*P* < 0.010).
SONG et al.^[42]^, 2019	NT-proBNP was associated with length of hospital stay (*P* < 0.001) and had an independent association with all-cause mortality (*P* = 0.003) and cardiovascular mortality (*P* = 0.002).
SPAMPINATO et al.^[36]^, 2020	NT-proBNP was independently associated with mortality when evaluated with two other biomarkers (*P* < 0.001).
	However, it was not associated with cerebrovascular events (*P* = 0.547) and new postoperative atrial fibrillation (*P* = 0.079).
TANAKA et al.^[37]^, 2021	NT-proBNP showed an independent association with the outcome composed of mortality and the need for postoperative hospitalization for HF (*P* = 0.030).
VERWIJMEREN et al.^[57]^, 2021	NT-proBNP was independently associated with acute kidney injury (*P* = 0.019).
VIKHOLM et al.^[58]^, 2014	NT-proBNP was associated with prolonged ICU stay (*P* < 0.010) and was independently associated with mortality (*P* = 0.010).
	There was no association with postoperative readmission (*P* = 0.270), postoperative infection (*P* = 0.440), acute kidney injury (*P* = 0.290), low cardiac output (*P* = 0.110), cerebrovascular events (*P* = 0.440), or bleeding (*P* = 0.930).
WANG et al.^[59]^, 2021	Preoperative NT-proBNP was independently associated with acute kidney injury (*P* < 0.001).
WEBER et al.^[38]^, 2006	NT-proBNP was not associated with mortality (*P* = 0.803) or post-surgery hospital readmission (*P* = 0.618).
WOZOLEK et al.^[60]^, 2022	NT-proBNP was associated with length of hospital stay (*P* = 0.010).
	There was no significant association between NT-proBNP mortality (*P* = 0.090), ICU length of stay (*P* = 0.124), acute kidney injury (*P* = 0.270), and low cardiac output (*P* = 0.090).
ZHAO et al.^[40]^, 2022	NT-proBNP had an independent association with mortality (*P* = 0.037) and cerebrovascular events (*P* = 0.037).

CI=confidence interval; EuroSCORE=European System for Cardiac Operative Risk Evaluation; HF=heart failure; IABP=intra-aortic balloon pump; LVEF=left ventricular ejection fraction; NT-proBNP=N-terminal pro-brain natriuretic peptide; OR=odds ratio

Additionally, one study assessed the association of preoperative NT-proBNP with postoperative circulating endothelial progenitor cells^[47]^, postoperative cardiac pump function^[35]^, and left ventricular diastolic dysfunction^[26]^ (Tables 1 and 2).

**Fig. 2:**
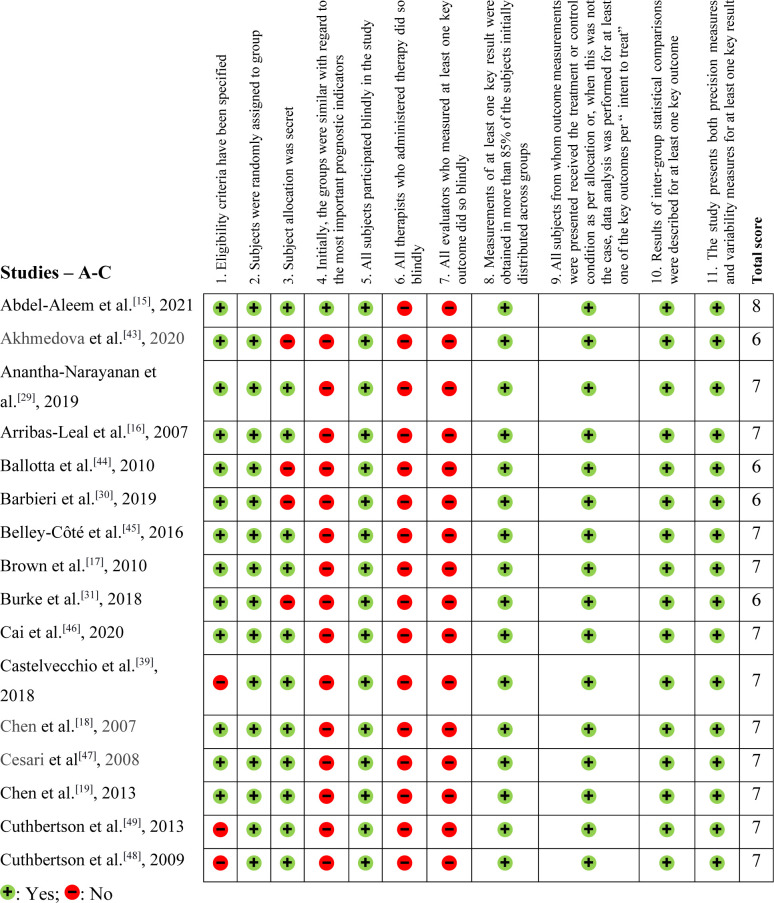
Physiotherapy Evidence Database (or PEDro) methodological assessment tool – Part 1 (studies starting from A to C).

**Fig. 3:**
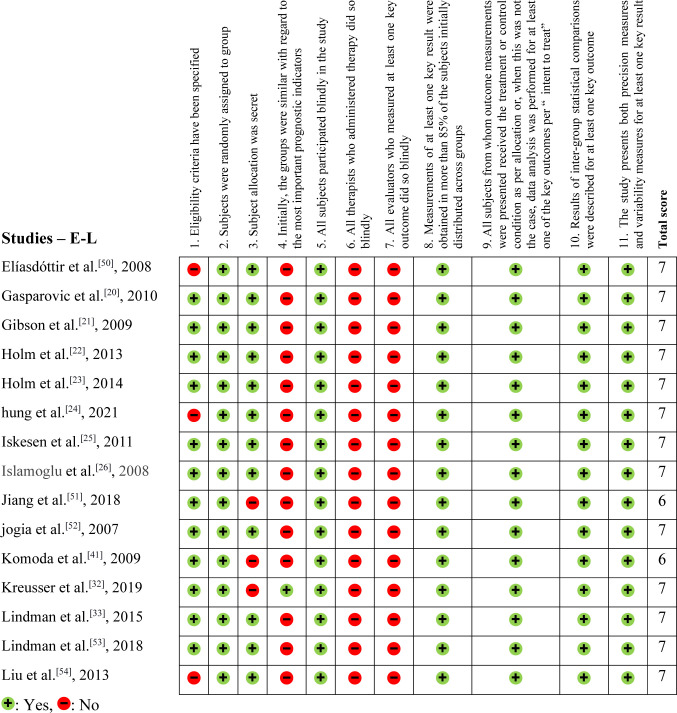
Physiotherapy Evidence Database (or PEDro) methodological assessment tool – Part 2 (studies starting from E to L).

**Fig. 4:**
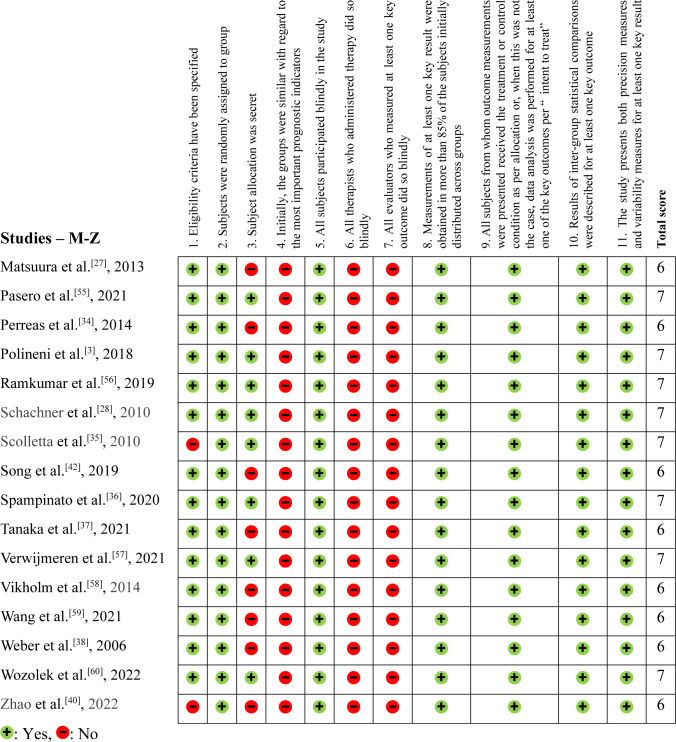
Physiotherapy Evidence Database (or PEDro) methodological assessment tool – Part 3 (studies starting from M to Z).

The methodological evaluation, demonstrated in Figures 2, 3, and 4, exposed that eight articles^[24,35,39,40,48–50,54]^ had problems with external validity according to the Physiotherapy Evidence Database (PEDro) scale (they did not present the inclusion or exclusion factors of their studies). According to the PEDro scale, 15 studies were negative for Question 3^[27,30-32,34,37,38,40-44,51,58,59]^, only two were positive for Question 4^[15,32]^, and none were positive for Questions 6 and 7.

The assessment of the risk of bias, inconsistency, indirectness, and imprecision using the PEDro and GRADE tools revealed that the overall quality of the evidence was generally not serious (Table 3). For new-onset AF, we observed a large association (odds ratio [OR] > 2.0 in at least two studies) and a very large effect on mortality (OR > 5.0). Additionally, a direct correlation was observed between NT-proBNP levels and mortality rates, as well as other adverse outcomes, including prolonged ICU stay, AKI, low cardiac output (LCO), new-onset AF, prolonged MV, cerebrovascular events, prolonged hospitalization, emergency reoperation, postoperative HF, and postoperative delirium.

**Table 3:** Grading of Recommendations, Assessment, Development and Evaluation.

Certainty assessment							№ of patients		Certainty
№ of studies	Study design	Risk of bias	Inconsistency	Indirectness	Imprecision	Other considerations	With postoperative complications	No postoperative complications	
**Mortality (follow-up: range 11 months to 10 years; assessed with: Number of Deaths)**									
33	Non-randomized studies	Not serious	Not serious	Not serious	Not serious	Very strong association directly proportional	1184/21275 (5.6%)	20091/21275 (94.4%)	⨁⨁⨁⨁
									High
**Prolonged ICU time (follow-up: range 2 months to 6 years; assessed with: Days)**									
16	Non-randomized studies	Not serious	Not serious	Not serious	Not serious	Directly proportional	759/7109 (10.7%)	6371/7109 (89.6%)	⨁⨁⨁⨁
									High
**Acute kidney injury (follow-up: range 2 months to 8 years; assessed with: Number of patients)**									
14	Non-randomized studies	Not serious	Not serious	Not serious	Not serious	Directly proportional	243/41351 (0.6%)	41108/41351 (99.4%)	⨁⨁⨁◯
									Moderate
**Low cardiac output (follow-up: range 1 year to 4 years; assessed with: Number of patients)**									
14	Non-randomized studies	Not serious	Not serious	Not serious	Not serious	Directly proportional	416/4662 (8.9%)	4246/4662 (91.1%)	⨁⨁⨁⨁
									High
**New atrial fibrillation (follow-up: range 6 months to 7 years; assessed with: Number of patients)**									
14	Non-randomized studies	Not serious	Not serious	Not serious	Not serious	Strong association directly proportional	1098/5409 (20.3%)	4311/5409 (79.7%)	⨁⨁⨁◯
									Moderate
**Prolonged time on mechanical ventilation (follow-up: range 2 months to 6 years; assessed with: Hours)**									
9	Non-randomized studies	Not serious	Not serious	Not serious	Not serious	Directly proportional	198/5657 (3.5%)	5459/5657 (96.5%)	⨁⨁⨁◯
									Moderate
**Cerebrovascular events (follow-up: range 17 months to 10 years; assessed with: Number of patients)**									
9	Non-randomized studies	Not serious	Not serious	Not serious	Not serious	Directly proportional	126/7065 (1.8%)	6942/7065 (98.3%)	⨁⨁⨁◯
									Moderate
**Prolonged hospitalization time (follow-up: range 11 months to 7.2 years; assessed with: Days)**									
8	Non-randomized studies	Not serious	Not serious	Not serious	Not serious	Directly proportional	140/2819 (5.0%)	2679/2819 (95.0%)	⨁⨁⨁◯
									Moderate
**Unscheduled hospital readmission related to heart problems (follow-up: range 18 months to 2 years; assessed with: Number of patients)**									
4	Non-randomized studies	Not serious	Not serious	Not serious	Not serious	None	249/1071 (23.2%)	822/1071 (76.8%)	⨁⨁◯◯
									Low
**Urgent reoperation due to bleeding (follow-up: range 17 months to 6 years; assessed with: Number of patients)**									
4	Non-randomized studies	Not serious	Not serious	Not serious	Not serious	Directly proportional	71/2794 (2.5%)	2723/2794 (97.5%)	⨁⨁⨁◯
									Moderate
**Acute myocardial infarction (follow-up: range 1 year to 17 months; assessed with: Number of patients)**									
3	Non-randomized studies	Not serious	Not serious	Not serious	Not serious	None	15/1267 (1.2%)	1252/1267 (98.8%)	⨁⨁◯◯
									Low
**Postoperative heart failure (follow-up: range 17 months to 7 years; assessed with: Number of patients)**									
3	Non-randomized studies	Not serious	Not serious	Not serious	Not serious	Directly proportional	265/3606 (7.3%)	3341/3606 (92.7%)	⨁⨁⨁◯
									Moderate
**Infection (follow-up: range 1 year to 4 years; assessed with: Number of patients)**									
3	Non-randomized studies	Not serious	Not serious	Not serious	Not serious	None	27/2763 (1.0%)	2736/2763 (99.0%)	⨁◯◯◯
									Very low
**Postoperative delirium (follow-up: range 3 months to 35 months; assessed with: Number of patients)**									
2	Non-randomized studies	Not serious	Not serious	Not serious	Not serious	Directly proportional	92/1018 (9.0%)	926/1018 (91.0%)	⨁⨁⨁◯
									Moderate

ICU=intensive care unit

For mortality and prolonged ICU stay, we found high-certainty evidence, suggesting that future research is unlikely to substantially impact our confidence in the effect estimate. For nine outcomes (AKI, LCO, new AF, prolonged MV time, cerebrovascular events, prolonged hospital stay, emergency reoperation for bleeding, postoperative HF, and postoperative delirium), the certainty of the evidence was moderate, suggesting that future research may significantly impact our confidence in the estimated effects. For unscheduled hospital readmission for cardiac reasons and new AMI, the certainty of the evidence was low or very low, indicating that future research is likely to substantially change our understanding of the effects. And there was one outcome (infection) with a very low level of evidence suggesting that any estimate of effect is very uncertain.

## DISCUSSION

In our review, we observed that, in those studies that evaluated mortality, approximately 7% of the patients died, which is a critical outcome for this patient profile. Twenty-five of the articles that assessed mortality^[3,17,22–24,28–30,32,34,36,37,39–42,44,45,48,50,51,53,54,56,58]^ agreed that high levels of preoperative NT-proBNP were related to higher mortality rates in cardiac surgical patients.

Holm et al.^[23]^ (2014), in their research, showed that patients with high preoperative levels of NT-proBNP were 9.94 times more likely (95% confidence interval [CI]: 1.01 – 98.9; *P* = 0.049) to die when compared to the group with low levels. This is in line with the work of Spampinato et al.^[36]^ (2020) who demonstrated that the same patient profile had a 7.26-fold increased risk (95% CI: 2.52 – 20.93; *P* < 0.001) of having this outcome. Polineni et al.^[3]^ (2018) reported that this patient profile was 5.43 times more likely (95% CI: 1.21 – 24.44; *P* = 0.027) of not surviving. This association was confirmed by all 16 studies^[17,22,28,30,32,33,37,39,40,42,45,48,51,54,56,58]^ that demonstrated an independent association between mortality and high NT-proBNP levels. Additionally, six other studies^[24,29,34,41,49,50]^ showed a significant association between these parameters.

On the other hand, Abdel-Aleem et al.^[15]^ (2021), Ballotta et al.^[44]^ (2010), Burke et al.^[31]^ (2018), Chen et al.^[19]^ (2013), Jogia et al.^[52]^ (2007), Weber et al.^[38]^ (2006), Lindiman et al.^[53]^ (2018), and Wozolek et al.^[60]^ (2022) failed to demonstrate this association. However, all these studies were developed with < 150 patients, except the last two, with 665 patients and 250 patients, respectively. Furthermore, none of the studies included a sample size calculation to ensure adequate statistical power. This omission raises concerns about the potential lack of representativeness of the study participants.

Another well-studied outcome was prolonged ICU stay, of which 16 of the studies included^[15,19,22,23,28,31,34,43,44,48,50–52,54,58,60]^ explained. Jiang et al.^[51]^ (2018) demonstrated a large-scale effect in their research, patients who had the highest NT-proBNP preoperatively were 2.87 times more likely (95% CI: 1.56 – 5.30; *P* = 0.001) to have long-term ICU stays. This was found also by Cuthbertson et al.^[48]^ (2009), who reported that higher levels of NT-proBNP at the preoperative time were independently associated with 1.03 more chances (95% CI: 1.01 – 1.05; *P* = 0.003) of longer than one day in the ICU. And it was corroborated by Liu et al.^[54]^ (2013) in their study of various cardiac surgeries, where they also showed an independent association of (*P* = 0.004) between NT-proBNP and this complication. Other six items^[23,28,31,44,50,58]^ followed this reasoning, demonstrating a significant association between the biomarker studied and this outcome, but they performed only univariate analyses in their studies, reducing the strength of the evidence.

The third most studied outcome was AKI, which 14 articles^[3,22,23,28,34,43–45,50,54,57–60]^ approached. Of the included studies, nine^[3,22,23,28,34,43,50,54,58]^ demonstrated a significant association between NT-proBNP levels and the development of postoperative AKI in univariate analysis. Belley-Côté et al.^[45]^ (2016) in their prospective cohort also showed an independent association between high pre-surgical levels of NT-proBNP and severe AKI (*P* = 0.030), which similarly occurred in the studies by Verwijmeren et al.^[57]^ (2021) e Wang et al.^[59]^ (2021).

The studies by Holm et al. (2013^[22]^, 2014^[23]^) revealed a very strong association between preoperative NT-proBNP levels and the development of LCO in multivariate analyses, with OR of 24.9 (95% CI: 2.9 - 214; *P* = 0.004) and 9.94 (95% CI: 1.01 - 98.9; *P* = 0.049), respectively. These findings underscore the high predictive power of preoperative NT-proBNP for this outcome, which may be attributed to its role as a biomarker of myocardial involvement. Of the other 12 articles^[3,15,18,43,44,48,50,52,54,55,58,60]^, ten studies^[3,18,43,44,48,50,52,54,55,58]^ agreed with this information.

Gibson et al.^[21]^ (2009) demonstrated an independent association between preoperative NT-proBNP and AF, bringing an increase of 3.12 times more chances (95% CI: 1.48 - 6.59; *P*=0.003) of developing new AF at the postoperative time when NT-proBNP levels were the highest preoperatively. Other six items^[3,20,25,27,34,48]^, of the 14 evaluated^[3,15,16,19–21,23,25,27,28,34,36,48,52]^, corroborated this association in their univariate analyses.

Nine studies^[15,22,28,43,44,48,51,52,54]^ evaluated the association between prolonged MV time and NT-proBNP, while seven^[22,28,44,48,51,52,54]^ agreed with the association between these parameters, but none of them performed a multivariate analysis to evaluate the possible confounding parameters of these associations, reducing the value of the evidence.

Regarding cerebrovascular events, five articles^[3,22,23,40,58]^ of nine^[3,22,23,28,36,40,44,51,58]^ agreed with the association between NT-proBNP and the aforementioned outcome. Of these, only Zhao et al.^[40]^ (2022) performed a multivariate analysis, demonstrating that high preoperative NT-proBNP levels were associated with 1,017 times more chances of developing them at the postoperative time.

The study of Cuthbertson et al.^[48]^ (2009) presented NT-proBNP levels as independent predictors of a hospital stay > 1 week (OR 1.070, *P* < 0.001), and this can be explained by the fact that the population evaluated was significantly higher. Liu et al.^[54]^ (2013) also found an independent association (*P* = 0.019) between these parameters. These findings were corroborated by the study by Song et al.^[42]^ (2019), which demonstrated in its univariate analysis that postoperative hospital stay was significantly longer in patients with NT-proBNP > 2080 pg/mL (*P* ≤ 0.001). These findings are supported by Wozolek et al.^[60]^ (2022) which also, in univariate analysis, presents a similar association (*P* = 0.010).

Tanaka et al.^[37]^ (2021) showed in their study that high levels of pre-surgical NT-proBNP were associated with rates of hospital readmission for cardiac causes, which were 1.5 times (95% CI: 1.03 – 2.17; *P* = 0.030) more likely to be readmitted to the hospital. The other three studies^[19,38,58]^ that evaluated this outcome did not find a statistically significant association.

None of the studies^[3,28,44,58]^ that evaluated emergency reoperation was able to express a statistically significant association, suggesting that preoperative NT-proBNP is not a good biomarker to predict this outcome.

AMI was a complication addressed in three articles in this review^[15,22,28]^. In the study by Abdel-Aleem et al.^[15]^ (2021), although 6% of the patients developed AMI in the postoperative period, there were no significant differences in preoperative NT-proBNP levels between patients with and without this complication (*P* = 0.397). The studies by Schachner et al.^[28]^ (2010) and Holm et al.^[22]^ (2013) also did not show a statistically significant association (*P* = 0.458 and *P* = 0.130, respectively).

The study by Tanaka et al.^[37]^ (2021) identified that the group with reduced NT-proBNP biomarker independently had a 1.5-fold lower risk of developing the composite outcome, including rehospitalization due to worsening HF (95% CI: 1.03 – 2.17; *P* = 0.03). Also, the study by Jiang et al.^[51]^ (2018) evaluated 2,978 patients and identified that preoperative NT-proBNP demonstrated an independent and significant association with postoperative severe heart failure (PSHF) in patients with coronary artery disease (CAD) and mitral regurgitation (*P* < 0.0001) and also with mitral stenosis (*P* = 0.047). In the multivariate analysis, NT-proBNP 855 ng/L emerged as an independent risk factor for PSHF in patients with CAD (adjusted OR 2.87; 95% CI: 1.56 – 5.30; *P* = 0.001).

Three articles^[3,28,58]^ evaluated the relationship between NT-proBNP and infections, from which only the study by Polineni et al.^[3]^ (2018), with a sample of 1,544 patients divided into tertiles and two groups based on the mean NT-proBNP value, demonstrated in the univariate analysis of the data an association between NT-proBNP and pneumonia (95% CI = 1,070 – 1,250); however, in the multivariate analysis, the association was not confirmed. Therefore, in none of the studies a relevant association was found between preoperative NT-proBNP values and the occurrence of infection in patients undergoing cardiac surgery.

Regarding postoperative delirium, only two articles studied the topic. Of these, only the study by Cai et al.^[46]^ (2020) demonstrated a significant and independent association (OR 1.240, 95% CI 1.010 – 1.520; *P* = 0.033) between preoperative NT-proBNP and the occurrence of this outcome.

In five studies, no association was found between pre-surgical NT-proBNP and any of the outcomes studied, those are: Abdel-Aleem et al.^[15]^ (2021), Lindiman et al.^[53]^ (2018), Arribas-Leal et al.^[16]^ (2007), Weber et al.^[38]^ (2006), and Chen et al.^[19]^ (2013). It is important to note that the absence of an association in certain studies underscores the heterogeneity of the surgical population and the potential influence of various factors on the relationship between NT-proBNP and postoperative outcomes. In addition, most studies evaluated surgeries used in different cardiac anatomical sites, which affects their recovery and complications, justifying possible disparities found. Another point worth noting was the scarcity of sample calculations to validate the representativeness of the studies.

In this systematic review, we found that the PEDro score was lower than expected due to the nature of the articles. Retrospective articles could not score positively on Question 3, for example. Likewise, these are not randomized studies, blinding patients and evaluators became unfeasible. Furthermore, the significant variability in NT-proBNP levels observed between the case and control groups made it challenging to achieve homogeneity among the study populations. This variability may have contributed to the high proportion of negative responses for Question 3 of the PEDro scale, which assesses the use of appropriate control groups.

### Limitations

There were no limitations to the current study.

## CONCLUSION

Pre-surgical NT-proBNP is a good independent biomarker to predict mortality, prolonged ICU stay, and LCO. Further studies are needed to evaluate its efficacy to independently predict postoperative AKI, new AF, cerebrovascular events, length of hospital stay, hospital readmission for cardiac causes, and postoperative delirium.

## Data Availability

The authors declare that the data supporting the findings of this study are available within the article.
